# High-resolution mapping of introduced buffel grass (*Cenchrus ciliaris*) in central Australia

**DOI:** 10.1007/s10661-026-15183-7

**Published:** 2026-04-03

**Authors:** Paul Box, Ian Leiper, Catherine Nano, Lauren Young, Valerie Caron, Sarah Gibson, Tracey Guest, Dale Cobban, Claire Treilibs, Jayne Brim Box

**Affiliations:** 1 Water Resources Division, Department of Lands, Planning and Environment, Northern Territory Government, Alice Springs, NT Australia; 2 Flora and Fauna Division, Department of Lands, Planning and Environment, Northern Territory Government, Darwin, NT Australia; 3 National Collections and Marine Infrastructure, Commonwealth Scientific and Industrial Research Organisation (CSIRO), Canberra, ACT Australia; 4 Uluru-Kata Tjuta National Park, Parks Australia, Yulara, NT Australia; 5 Water Resources Division, Department of Lands, Planning and Environment, Northern Territory Government, Palmerston, NT Australia

**Keywords:** Weeds, Time series, Singular value decomposition, Remote sensing, Invasive species

## Abstract

Buffel grass (*Cenchrus ciliaris*) is an introduced pastoral grass that threatens arid ecosystems worldwide. Its ability to rapidly colonise new areas, outcompete native plants, invade a diversity of habitats and fuel fires make it a disaster for native biodiversity, infrastructure and remote communities. This species was implicated in the fires that devastated Lahaina, Hawaiʻi in 2023, and in Australia, it is one of the most significant environmental threats to semi-arid rangelands. The management of buffel grass requires detailed knowledge of its current spatial distribution, information currently lacking for most of inland Australia. We modelled and mapped buffel grass occurrence at two sites in central Australia by applying singular value decomposition to a time series of vegetation indices derived from Sentinel-2 satellite imagery. In both study areas, buffel grass could be modelled/mapped with high accuracy (≥ 85%) at the native resolution of the satellite imagery (10 m). Our study areas were much larger (up to 2800 km^2^) than previous attempts to map buffel grass in both Australia and overseas, and our model accuracies were either comparable or higher than previous efforts. The buffel grass maps can aid resource managers and practitioners in their control and monitoring efforts, and our method uses free, open-source software, making it more cost effective than methods using drones and/or aerial surveys.

## Introduction

Native to parts of East Africa, the Middle East and southern Asia, buffel grass (*Cenchrus ciliaris*) is an introduced pastoral grass that poses a significant risk to dryland ecosystems worldwide (IPBES, 2023). It is drought resistant, fast growing, forms dense stands, can withstand heavy grazing and is highly palatable, hence its popularity for livestock production (Friedel et al., 2006) and erosion control (Humphries et al., 1991). Buffel grass can rapidly colonise new areas, outcompete native plants, invade a wide range of habitats and carry fire, attributes that contribute to severe impacts on native biodiversity (Franks, 2002; Lawson et al., 2004; Wright et al., 2020).

Worldwide, awareness of the negative impacts of buffel grass on native species and ecosystems has grown considerably in recent years. In the Sonoran Desert in the southwestern United States, buffel grass was introduced in the 1940 s for cattle fodder and erosion control (Hovanes et al., 2023) and today outcompetes native plant species, including the saguaro cactus, and fuels frequent and intense fires in areas where fire was previously rare (McDonald & McPherson, 2011; Rodriguez-Rodriguez et al., 2017). Recent models, based partly on predicted climate change parameters, suggest that nearly 50% of Mexico is highly suitable to buffel invasion (Siller-Clavel et al., 2022), including semi-arid and arid lands with high plant endemism (Riemann & Ezcurra, 2005). In Hawaiʻi, wildfires fuelled by invasive grasses are increasing (Trauernicht et al., 2015), and buffel grass was implicated as a causal factor in the deadly fires in Lahaina in 2023. Areas of Hawaiʻi invaded by buffel and other grasses are resilient to change post-fire, including recolonization by native grasses (Yelenik et al., 2024).

Buffel grass was first introduced to Australia in the 1870 s by cameleers. Its expansion across arid Australia was limited until the 1970 s, when it was deliberately planted for erosion control, dust suppression and improved livestock production (Friedel et al., 2006). Today, buffel grass is considered a key threat to native biodiversity across arid Australia (Read et al., 2020; Ryan-Colton et al., 2024). Buffel grass outcompetes ground-storey plant species and limits growth and recruitment in shrubs and trees (Franks, 2002). Heavily infested sites face a high risk of frequent and severe fire due to increased ground fuel loads and connectivity (Miller et al., 2010). This greatly disadvantages trees and shrubs through stem kill, canopy loss or outright death, and habitats can be rapidly transformed from species-rich shrubland to low diversity grassland under these changed conditions (e.g. Schlesinger et al., 2013). Negative flow-on effects for fauna are also well documented, with a loss of habitat and foraging opportunities having particularly severe impacts on woodland birds (Young and Schleisenger, 2015) and open-ground foraging reptiles (McKinney et al., 2014). Risks to infrastructure and housing from wildfire have also increased over the past decade due to the spread of buffel grass into conservation areas and regional towns.

Aboriginal people recognise the potential threat of buffel grass to culture and the environment (Read et al., 2020), and in the local Pitjantjatjara language, buffel grass is referred to as *mamu tjanpi* or ‘devil grass’ (Alinytjara Wilurara Landscape Board, 2023). Buffel grass can reduce the diversity of native plants traditionally used as bush foods and medicine, damage sacred sites and impede the transfer of intergenerational cultural knowledge (Read et al., 2020). Buffel grass removal was identified as a priority by Arrernte Traditional Owners as a key step to restore and protect sacred waterholes and important plant communities (Caron et al., 2021). People living in remote Aboriginal communities are also at risk from intense fires caused by buffel grass (Bowman, 2018). Managing buffel grass is therefore important to not only protect biodiversity, but also communities and cultural heritage.

Effective buffel grass management in central Australia requires detailed knowledge of its spatial distribution. To date, buffel grass has been successfully mapped in small areas (Elkind et al., 2019; Marshall et al., 2012; Wallace et al., 2016), but not across larger landscapes (e.g. > 1000 km^2^). Methods for differentiating buffel grass from native grasses using remote sensing have relied on capturing satellite images after rain events, when buffel grass ‘greens up’ relatively quickly and has a spectral signature distinguishable from native grasses (Wallace et al., 2016). This approach is limited because the spectral signal of buffel grass can change quickly as it dries out and when it is dry, its spectral signal is very similar to other dry vegetation, including native grasses. Rainfall events in central Australia are often of short duration (i.e. lasting a few days) and can be highly localised, so it can be difficult to track individual rainfall events and acquire suitable satellite imagery.

Here, we used a recently developed mapping technique (Brim Box et al., 2022) that is not subject to the above limitations, to produce high-resolution maps of buffel grass distribution in central Australia. The technique was developed to map groundwater-dependent ecosystems (GDEs) in central Australia, and it has not yet been applied to grassy dryland systems. We predicted that buffel grass, like GDEs, could be detected from signals in a time series of vegetation indices derived from high-resolution satellite imagery. We expected the distinct temporal signal of buffel grass could be distinguished from native grasses and other vegetation types. We applied the technique to two contrasting areas in the central Australian landscape to assess its accuracy and reliability across major regional environmental gradients (climate, geomorphology, soil, elevation and vegetation).

## Methods

### Study areas

**Fig. 1:**
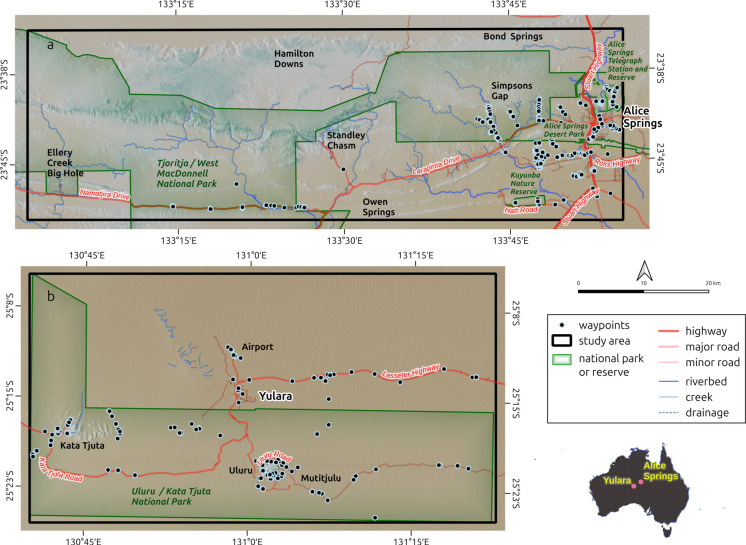
**a** and **b** Study areas: Tjoritja/West MacDonnell National Park (TWMNP) (**a**) and Uluru-Kata Tjuta National Park (UKTNP) (**b**)

Two distinct study areas within central Australia were selected—the eastern half of the Tjoritja/West MacDonnell National Park (TWMNP) and the Alice Springs municipality, and the Uluru-Kata Tjuta National Park (UKTNP) (Fig. 1a and b). These two study areas represent contrasting and highly characteristic central Australian ecosystems, allowing for the methodology to be applied and assessed across a variety of land and vegetation types encountered throughout the broader region. While their topography, localised hydrography and ecoregions differ, they are relatively close geographically, subject to broadly similar climate histories (e.g. droughts, el Niño), and are roughly the same size (~ 2800 km^2^). Both study areas were assessed using the same satellite platform (Sentinel-2), spatial resolution (10 m) and similar time frames (2015–2024/2025).

### Tjoritja/West MacDonnell National Park (TWMNP)

**Fig. 2:**
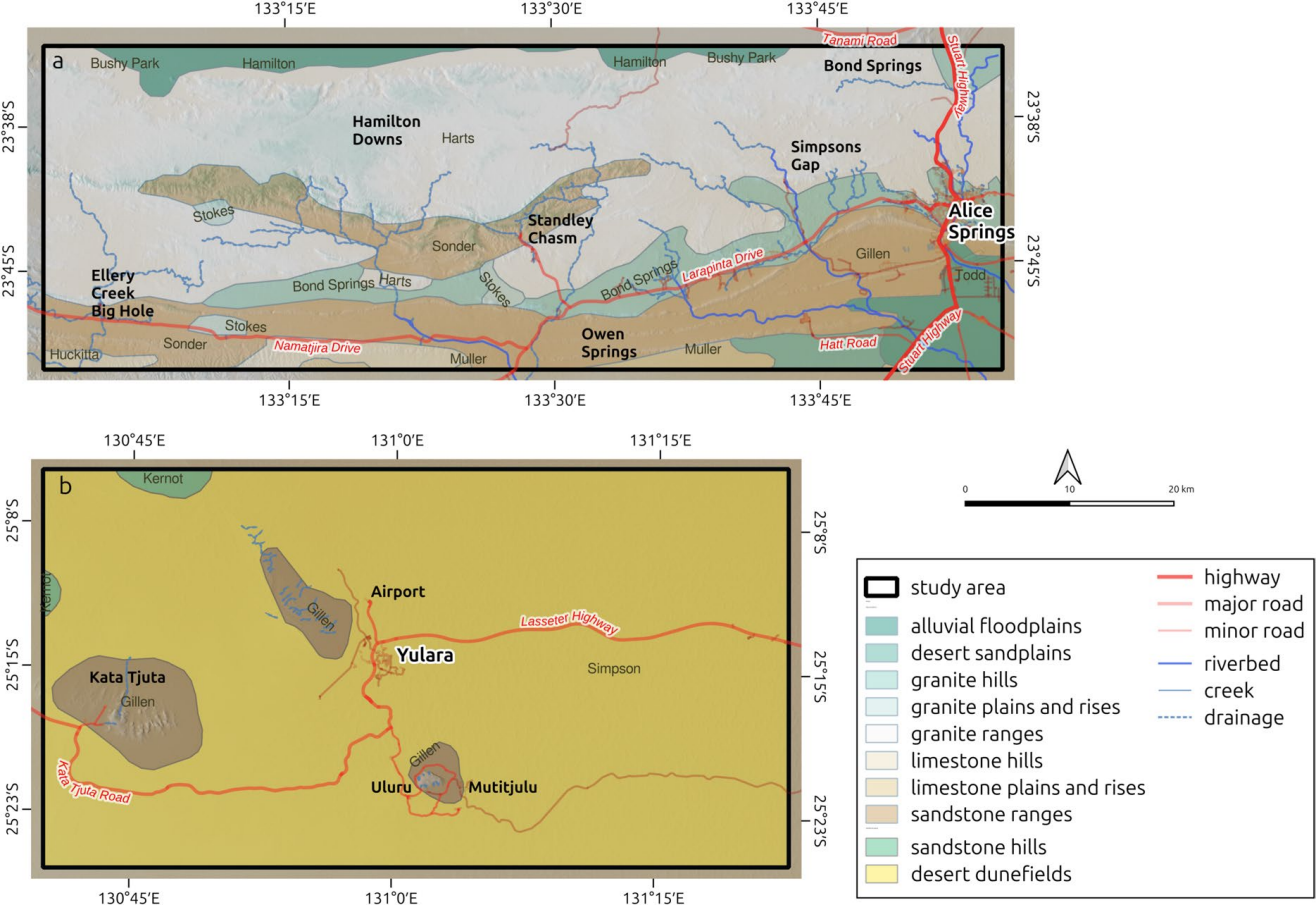
**a** and **b** Landscape classes within the Tjoritja/West MacDonnell National Park (TWMNP) (**a**) and Uluru-Kata Tjuta National Park (UKTNP) (**b**) study areas. Information on landscape classes for the two study areas is based on Perry (2010) and DLPE (2018, https://nrmaps.nt.gov.au)

The TWMNP study area covers approximately 2860 km^2^, including the eastern half of the national park and the Alice Springs municipality (Fig. 1a). This study area incorporates multiple land tenures, including conservation reserves, pastoral leases, Aboriginal land trusts and vacant crown land. Multiple landscape types are represented and include alluvial floodplains; desert sandplains; granite plains, rises and hills; sandstone/quartzite hills and rugged ranges; and limestone plains, rises and hills. This variability has been summarised and mapped as ‘Land Systems’ (Perry, 2010) (Fig. 2a and b; Table 1).

**Table 1:** Land systems in the Tjoritja/West MacDonnell National Park (TWMNP) and Uluru-Kata Tjuta National Park (UKTNP) study areas. Information on land systems for the two study areas is based on Perry (2010) and DLPE (2018, https://nrmaps.nt.gov.au). Area is the total land system area within the study areas

Study area	Area (km^2^)	Land system	Geomorphic Zone	Class	Class description
TWMNP	1541	Harts	Burt Plain	Granite ranges	Rugged mountain ranges on gneiss, schist and granite; outcrop with shallow, gritty and stony soils
	493	Gillen	MacDonnell Ranges	Sandstone ranges	Rugged ranges on quartzite, sandstone and conglomerate; outcrop with shallow, stony sandy soils
	267	Bond Springs	Burt Plain	Granite hills	Low hills and hills mostly on granite, gneiss, rhyolite and some schist; common rock outcrop and surface stone with shallow gritty or stony soils
	219	Sonder	Burt Plain	Sandstone ranges	Rugged ranges on quartzite, sandstone and conglomerate; outcrop with shallow, stony sandy soils
	94	Todd	Finke	Alluvial floodplains	Alluvial floodplains, swamps, drainage depressions and alluvial fans; sandy, silty and clay soils on Quaternary alluvium
	45	Hamilton	Burt Plain	Alluvial floodplains	Alluvial floodplains, swamps, drainage depressions and alluvial fans; sandy, silty and clay soils on Quaternary alluvium
	42	Pertnjara	MacDonnell Ranges	Limestone hills	Dissected hills on Cambrian limestone and dolomite; shallow soils with rock outcrop
	32	Ewaninga	MacDonnell Ranges	Desert sandplains	Level to undulating sandplains with red sands
	25	Stokes	MacDonnell Ranges	Granite plains and rises	Gently undulating to undulating plains with rises and low hills on granite, schist, gneiss (deeply weathered in places); coarse grained sandy, earthy and texture contrast soils
	35	Muller	MacDonnell Ranges	Limestone plains and rises	Plains, rises and plateaux on weathered and unweathered Cambrian limestone, dolomite, chalcedony, shale, sandstone and siltstone with associated sand sheets; sandy and earth soils
	9	Bushy Park	Tanami	Alluvial floodplains	Alluvial floodplains, swamps, drainage depressions and alluvial fans; sandy, silty and clay soils on Quaternary alluvium
	6	Huckitta	Burt Plain	Limestone hills	Dissected hills on Cambrian limestone and dolomite; shallow soils with rock outcrop
UKTNP	2544	Simpson	Tanami	Desert dunefields	Dunefields with parallel linear dunes, reticulate dunes and irregular or aligned short dunes; red sands
	175	Gillen	MacDonnell Ranges	Sandstone ranges	Rugged ranges on quartzite, sandstone and conglomerate; outcrop with shallow, stony sandy soils
	24	Kernot	Great Sandy Desert	Sandstone hills	Low hills, hills and stony plateaux on sandstone, siltstone, quartzite and conglomerate (deeply weathered in places); outcrop with shallow stony soils

A diversity of vegetation types occurs in the TWMNP study area. To the east, the Todd River is lined with river red gum (*Eucalyptus camaldulensis*) woodland and is now heavily infested with buffel grass and couch grass. Similarly, the adjacent ironwood (*Acacia estrophiolata*) floodplains are severely degraded by buffel grass. Buffel grass is present in associated coolabah swamps, but to date its impact on these sites is less severe. Similarly, saline areas with saltbush shrublands (*Atriplex spp*.) have so far withstood heavy infestation. The gneiss and granite ranges and hills support mulga (*Acacia aneura sens. lat*.), witchetty bush (*Acacia kempeana*), *Senna* spp. shrublands and spinifex (*Triodia* spp*.*) hummock grasslands. These relatively nutrient-rich land types are heavily infested with buffel grass and the shrublands are becoming increasingly degraded by fire. The Gillen quartzite and sandstone ridges are, in general, less impacted by buffel grass; however, significant incursions do exist throughout this land system.

Within the TWMNP, buffel grass forms very dense stands in creeks and drainage lines as well as along major riverbanks and on floodplains. Very few habitats are now free of buffel grass, with recent incursions observed at sites previously thought resistant (e.g. dolomite hills and high-quartzite mountain peaks). Buffel grass infestation contributed significantly to the two most recent wildfires in the park, both of which caused significant damage to biodiversity values and to park infrastructure. In 2019, uncontrolled wildfires burned a 100 km swath from Mount Sonder in the west to Jay Creek in the east. In 2023, wildfires burned over 100,000 ha of the park (about 20% of the total area).

### Uluru-Kata Tjuta National Park (UKTNP)

The UKTNP study area covers approximately 2735 km^2^, including the entire national park and the township of Yulara. This study area is characterised by parallel, reticulate and aligned short dunes and red sands of the Simpson land system, and by the arkose of Uluru and boulder conglomerate of Kata Tjuta, which represent small islands of the Gillen land system (Perry, 2010).

The landscape is dominated by sandplains and dunefields characterised by spinifex (*Triodia spp.*) hummock grassland with low scattered shrubs and tall desert oak (*Allocasuarina decaisneana*) stands (Central Land Council 2015). The dune upper slopes and crests support a specialised flora of shrubs and herbs. These habitats often evade fire and are characterised by loose mobile sands. Areas of mallee (*Eucalyptus* spp.) shrubland/woodland occur in the sandplain landforms, providing important habitat for fauna. There is evidence of increasing buffel grass presence, especially directly under tree canopies where there is greater shade and higher soil resources.

The rocky areas, including Uluru and Kata Tjuta, are unvegetated apart from the restricted occurrences of specialised species in rock crevices. Waterholes and soaks associated with Uluru and Kata Tjuta support rare and restricted plant species that are dependent on perennial moisture and occur nowhere else in the park. The run-on areas at the base of these major rock formations support bloodwood (*Corymbia*) woodlands with *Acacia* shrubs and numerous native perennial grass species. These wooded areas are now heavily infested with buffel grass and are subject to ongoing weed management. Similarly, rocky creeks lined with river red gum (*Eucalyptus camaldulensis*) and the floodouts around Kata Tjuta are heavily infested with buffel grass, resulting in increased fire frequency and severity in those habitats. Large stands of mulga (*Acacia aneura sens. lat*.) and other *Acacia* species dominate the sandy red earth plains surrounding Uluru and Kata Tjuta. These shrublands also occur throughout the park in dune swales and on open plains. Currently, buffel grass occurs in relatively low density in these habitats, but incursions have been recorded.

### Satellite data and vegetation indices

Our study used data from the Copernicus Sentinel-2 mission, a constellation of satellites, each with a high-resolution multispectral imager. Sentinel-2A and 2B were launched in 2015 and 2017, respectively, each with a visitation rate of ten days and a combined effective visitation rate of every five days from 2017 onwards. Spectral data from visible and near infrared (NIR) wavelengths are available at a spatial resolution of 10 m, and from shortwave infrared (SWIR) wavelengths at a 20-m spatial resolution.

Sentinel-2 imagery acquired between December 2015 and February 2025 was compiled for TWMNP, and between December 2015 and August 2024 for UKTNP. Imagery was obtained via the open-source Digital Earth Australia (DEA) Open Data Cube (https://www.ga.gov.au/scientific-topics/dea/about/open-data-cube; Lewis et al., 2017). We utilised their Analysis Ready Data because all processing, validation and corrections are adjusted explicitly for Australian conditions by Geoscience Australia, including corrections to surface reflectance (https://knowledge.dea.ga.gov.au/notebooks/Beginners_guide/02_DEA/).

Because both study areas spanned multiple Sentinel-2 tiles, image mosaics were created for each unique acquisition date, with a spatial resolution of 10 m. Images were included in the mosaics if they contained less than 3% cloud cover and image mosaics were retained for further analysis if they contained more than 90% non-null values (i.e. > 90% of the study area contained data). This yielded final time series of 391 image mosaics for TWMNP and 185 image mosaics for UKTNP. These time series represent data cubes—a three-dimensional array with every value in the array indexed to a location, with two dimensions representing northing and easting, respectively, and the third dimension representing time (the acquisition date of each image).

Vegetation indices are used to highlight spectral features in satellite data that relate to the photosynthetic activity and water content of plants. Four commonly used vegetation indices were utilised in this study including the Enhanced Vegetation Index (EVI; Wang et al., 2023), the Normalised Burn Ratio (NBR; Key & Benson, 2006), the Normalised Difference Vegetation Index (NDVI; Rouse et al., 1974; Tucker, 1979) and the Normalised Difference Water Index (NDWI; Gao, 1996). The first three vegetation indices are commonly used to examine ecological change over time, particularly at a landscape-level, and the fourth index (NBR) was included in this study because it encodes fire history. The phenology of buffel grass is influenced by rainfall and fire history.

The four vegetation indices were calculated (Table 2) for each study area, creating a total of eight data cubes. Each data cube was reshaped into a two-dimensional spatiotemporal matrix for each vegetation index, with each row containing the contents of the Sentinel-2 mosaic on its acquisition date, and each column representing the vegetation index values for each pixel through time. This matrix can simultaneously be conceived as a stack of image mosaics for a given time period or a collection of time series for a given space.

**Table 2:** Vegetation indices calculated using Sentinel-2 reflectance data. G is a gain factor (G = 2.5), C1 and C2 are coefficients to correct for aerosol scattering (C1 = 6, C2 = 7.5); L is a coefficient to correct for soil background (L = 1) and SWIR1 indicates that the first Sentinel-2 shortwave infrared band was used

Index	Formulation	Reference
Enhanced Vegetation Index (EVI)	$\frac{G*\left(NIR-RED\right)}{\left(NIR+C1*RED-C2*BLUE+L\right)}$	Wang et al., 2023
Normalised Burn Ratio (NBR)	$\frac{\left(NIR-SWIR1\right)}{\left(NIR+SWIR1\right)}$	Key & Benson, 2006
Normalised Difference Vegetation Index (NDVI)	$\frac{\left(NIR-RED\right)}{\left(NIR+RED\right)}$	Rouse et al., 1974, Tucker, 1979
Normalised Difference Water Index (NDWI)	$\frac{\left(GREEN-NIR\right)}{\left(GREEN+NIR\right)}$	Gao, 1996

### Singular value decomposition

We used singular value decomposition (SVD) to extract the dominant temporal patterns from the satellite data. SVD is a mathematical technique (matrix decomposition) that summarises large datasets into orthogonal components, often referred to as eigenvectors. SVD can efficiently partition data variance and performs well in remote sensing applications (Brim Box et al., 2022; Phillips et al., 2009). Because our dataset was arranged with image dates as rows and individual pixels as columns, the SVD distinguished temporal variation from spatial variation. The resulting components described how vegetation indices change over time and across the landscape. Only the leading components were retained for subsequent analysis, as they captured most of the variation in the dataset (i.e. the broadest and most meaningful trends). A more detailed description of the mathematical implementation is provided in Brim Box et al. (2022).

By examining the leading components, we identified the principal modes of variation in the time series—those most likely to correspond to vegetation responses such as growth, greening and senescence following rainfall or fire. The first component represented the greatest source of variation (up to 61% in each study area), which typically reflected the general median condition and provided a reasonable estimate of the vegetation type or cover expected over time, given variations in rainfall and topography. The remaining components described deviations away from this global median model expressed in the first component. Collectively, these SVD component values characterised aspects of the phenological or behavioural history of the vegetation indices at each pixel (i.e. the shape of the curve if plotted on a graph). Combinations of similar values indicated similar temporal histories, whereas differing combinations indicated distinct histories.

This is a computationally intensive process but is tractable with what is considered a high-end gaming computer as of the publication date of this paper. We used an HP Omen desktop with an Intel i9 13900 K 32 core processor, 64GB of native memory, and 200GB of swap space enabled, using Ubuntu 24 as the OS. Each data cube in TWMNP (the larger of the two sites) contained 11 billion elements for the four vegetation indices, each of which was processed in about 15 min using the sklearn 1.3.0 randomised SVD function and scikit 1.11.1 (Li et al., 2017; Pedregosa et al., 2011).

### Modelling

We hypothesised that given the distinctive phenological response of buffel grass to rainfall, it should be possible to distinguish buffel grass from other vegetation types using temporal patterns extracted from the time series data via SVD. Due to its extensive (but shallow) root system, buffel grass can respond rapidly to rain events. Consequently, soon after rainfall, buffel grass ‘spikes’ in greenness value (e.g. NDVI) and obtains higher values than native grasses (Gerst et al., 2021). It also forms dense mats, and thus, vegetation index values (e.g. NDVI) remain higher for longer than native grasses. Specifically, we expected that there was a unique combination of SVD eigenvector values that were reliably and uniquely consistent with the presence of buffel grass. Given the top six eigenvectors of each vegetation index described ~ 90% of the total variance in their respective matrices, these six eigenvectors were retained in the analysis, giving 24 input variables for each study area.

For model training and evaluation purposes, field surveys were undertaken and existing data collated to produce datasets on the occurrence and absence of buffel grass in each study area. A total of 480 and 184 unique waypoints were compiled for TWMNP and UKTNP, respectively. Waypoints were chosen based on a colour-composite map derived from a SVD of the NDVI time series for each study area, with colours representing similar temporal histories, as described above. We used these maps to target pixels thought to contain buffel grass, as well as pixels without buffel grass present. Thus, waypoints were collected across land systems and vegetation types within each study area. In the TWMNP, waypoints were collected from the major land systems that together constituted 88% of the study area. Land systems not sampled were roadless/inaccessible or occurred in very small areas. In UKTNP, waypoints were collected from two of the three land systems, which constituted 99% of the study area. In both study areas, waypoints were then classified as containing ‘buffel grass’ or as ‘buffel-grass free’. Additional site attributes were also recorded, including dominant upper-, mid- and groundcover species, landform type and evidence of recent grazing and/or fire.

We applied the same modelling procedure described in Brim Box et al. (2022). Logistic regression models were used to evaluate whether sites could be accurately classified as containing buffel grass, based on the SVD-derived components, and to map buffel grass probabilities in each study area. Logistic regression employed a forward stepwise selection guided by the Bayesian Information Criterion (BIC) to retain only components that significantly improved model fit. The BIC was used rather than the more widely known corrected Akaike’s Information Criterion (AIC_c_) because BIC penalises models more than AIC_c_ for having more parameters (Burnham & Anderson, 2004), and our goal was to select the most parsimonious models possible.

Given the nonlinearity of the data and the use of a novel technique (SVD) to map buffel grass, we also applied neural network models to interrogate the same data sets. Together, logistic regression and neural nets are commonly used to model and compare nonlinear datasets (e.g. Issitt et al., 2022). The neural net models incorporated all 24 eigenvectors as inputs and were configured with a single hidden layer of three nodes using a hyperbolic-tangent activation function to account for potential non-linearity in the data. Two-thirds of the observations were used for training and one-third for validation in both model types, and each model was bootstrapped ten times to obtain average performance statistics. Model accuracy was assessed using the standard metrics of sensitivity, specificity and overall accuracy, defined as the proportions of correctly classified presences and absences relative to the total number of observations. Receiver operating characteristic (ROC) curves were generated and the area under curve (AUC) provided an overall measure of predictive success. All statistical analyses used JMP 18 (SAS Institute, Cary, NC, USA).

### Buffel grass probability map

As described in Brim Box et al. (2022), we used logistic regression models to estimate the probability of buffel grass in both study areas. Logistic regressions were used over neural nets because neural networks are highly parameterised and a formula for the parametric form to estimate buffel grass presence required 79 parameters, compared to a maximum of 24 parameters for the logistic regression models. We estimated the probability of buffel grass presence for every pixel in each study area using the standard logistic transformation:

$$P(Buffel)\:=\:1/(1\:+\:e^{Lin(n)}),$$

where Lin(n) = β_0_ + ∑β_i_X_i_ with β_i_ being the parameter estimates from the logistic regression and X_i_ the retained eigenvalues. This computation produced a continuous surface of probability from 0 (very unlikely) to 1 (very likely), which was subsequently visualised as a map of buffel grass occurrence.

## Results

### Modelling

Neural network model results were similar to the logistic regression results in both study areas (Table 3). There was very little difference in model accuracy, as assessed by sensitivity, specificity, overall accuracy and AUC, between the neural net and logistic regression models. In addition, the consistency between training and validation sets within and across models indicated that both model types were robust and not over-fitted. These results supported our use of the logistic regression models, for the reasons described earlier, to estimate buffel probabilities in both study areas.

**Table 3:** Bootstrapped logistic regression (LR) and neural network (NN) model results for the two study areas (Tjoritja/West MacDonnell National Park (TWMNP) and Uluru-Kata Tjuta National Park (UKTNP)). Models mean (X̅), and standard error (SE) values are based on ten bootstraps; *AUC* =area under curve of the receiver operating characteristic curve

Study area	Model	Specificity (X̅ + SE)	Sensitivity (X̅ + SE)		Accuracy (X̅ + SE)	AUC (X̅ + SE)
TWMNP	LR (training)	84.91 ± 0.43		84.44 ± 0.49	0.85 ± 0.003	0.93 ± 0.003
	LR (validation)	86.70 ± 1.48		85.89 ± 1.16	0.86 ± 0.004	0.94 ± 0.006
	NN (training)	86.11 ± 0.69		84.70 ± 0.86	0.85 ± 0.004	0.93 ± 0.004
	NN (validation)	83.53 ± 1.53		80.59 ± 1.80	0.82 ± 0.013	0.89 ± 0.013
UKTNP	LR (training)	94.1 ± 0.62		89.90 ± 1.12	0.93 ± 0.008	0.97 ± 0.003
	LR (validation)	95.7 ± 0.79		92.90 ± 0.72	0.95 ± 0.007	0.98 ± 0.002
	NN (training)	95.5 ± 1.11		90.80 ± 2.08	0.94 ± 0.012	0.98 ± 0.005
	NN (validation)	89.1 ± 2.00		85.10 ± 2.31	0.88 ± 0.012	0.95 ± 0.007

SVD components derived from multiple vegetation indices produced the most powerful models (i.e. better ability to correctly classify sites with buffel grass present) than using components from a single vegetation index. The final logistic regression model for TWMNP included eigenvector values derived from all four vegetation indices, while the final model for UKTNP included values from three of the four indices. The average model accuracy was 85% for TWMNP and 94% for UKTNP, while the average bootstrapped AUC of the ROC curve was 0.93 for TWMNP and 0.97 for UKTNP (Table 3), indicating strong model performance.

### Buffel grass probability map

To generate buffel grass probability maps, we applied the same logistic regression approach described in Brim Box et al. (2022), using SVD-derived components as predictors. The following equations (best-fitting logistic regression models) were applied for each study area, respectively:

$$TWMNP\;buffel\;grass\;probability\;=\;-\;2.92\;-\;1246(ndvi_3)\;+\;6036(ndvi_4)\;-\;3210(ndvi5)\;+\;21049(evi_1)\;-\;6328(evi_3)\;-\;7503(evi_5)\;-\;10808(ndwi_2)\;+\;1564(ndwi_6)\;+\;11760(nbr_1)\;+\;72.5\;(nbr_2)\;-\;6105\;(nbr_3)\;+\;2027(nbr_6)$$

$$UKTNP\;buffel\;grass\;probability\:=\:-\;6.48\:+\:2292(ndvi_3)\:+\:8902(evi_5)\:+\:7710(ndwi_2)\;\:-\;10,641(ndwi_4)$$

where subscripts refer to the component of each vegetation index. Each pixel within the study areas was thus assigned a probability value ranging from zero (very low probability of that pixel containing buffel grass) to one (very high probability of that pixel containing buffel grass).

**Fig. 3:**
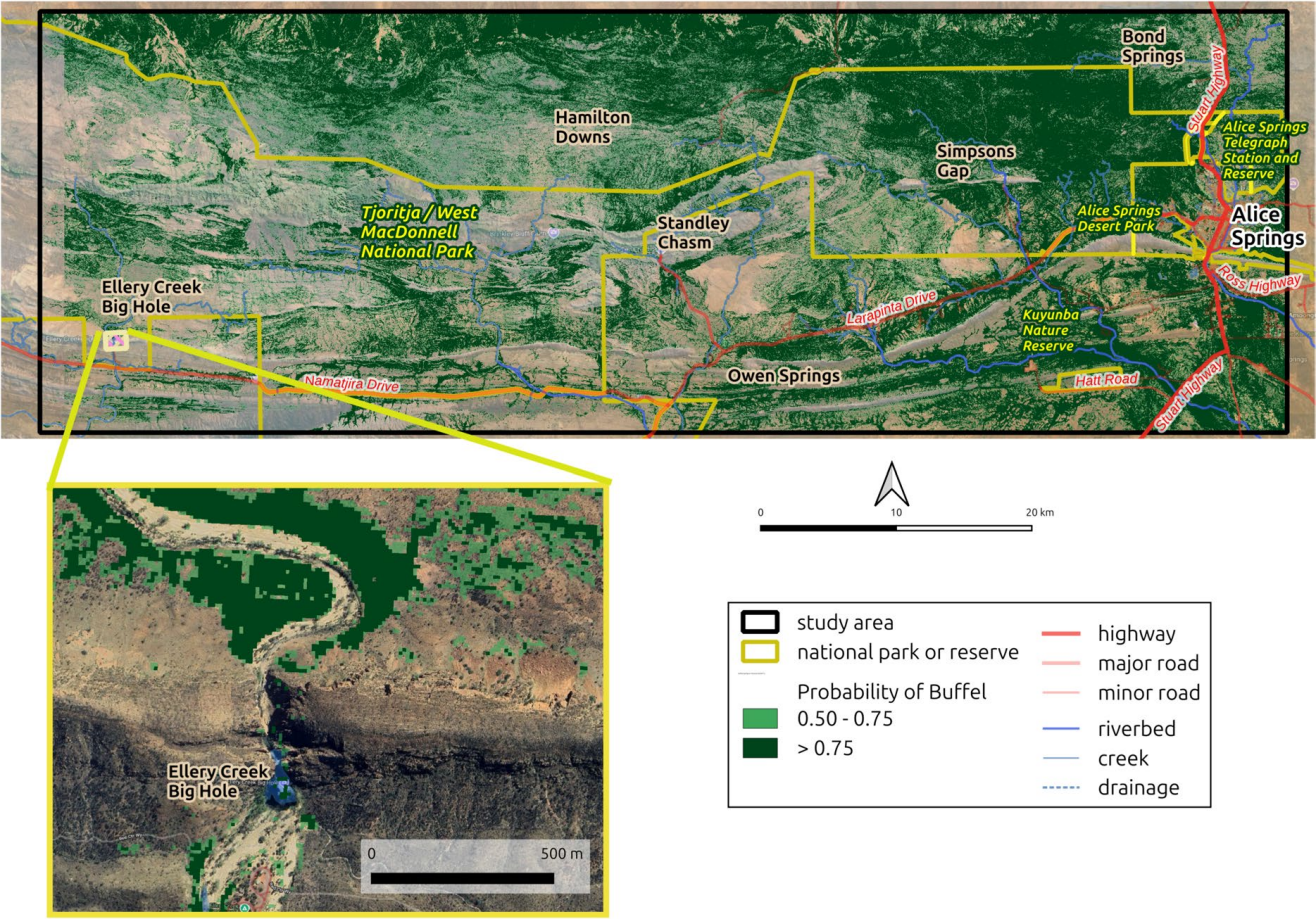
Buffel grass probability map for Tjoritja/West MacDonnell National Park (TWMNP). Imagery © 2026 Google Earth

**Fig. 4:**
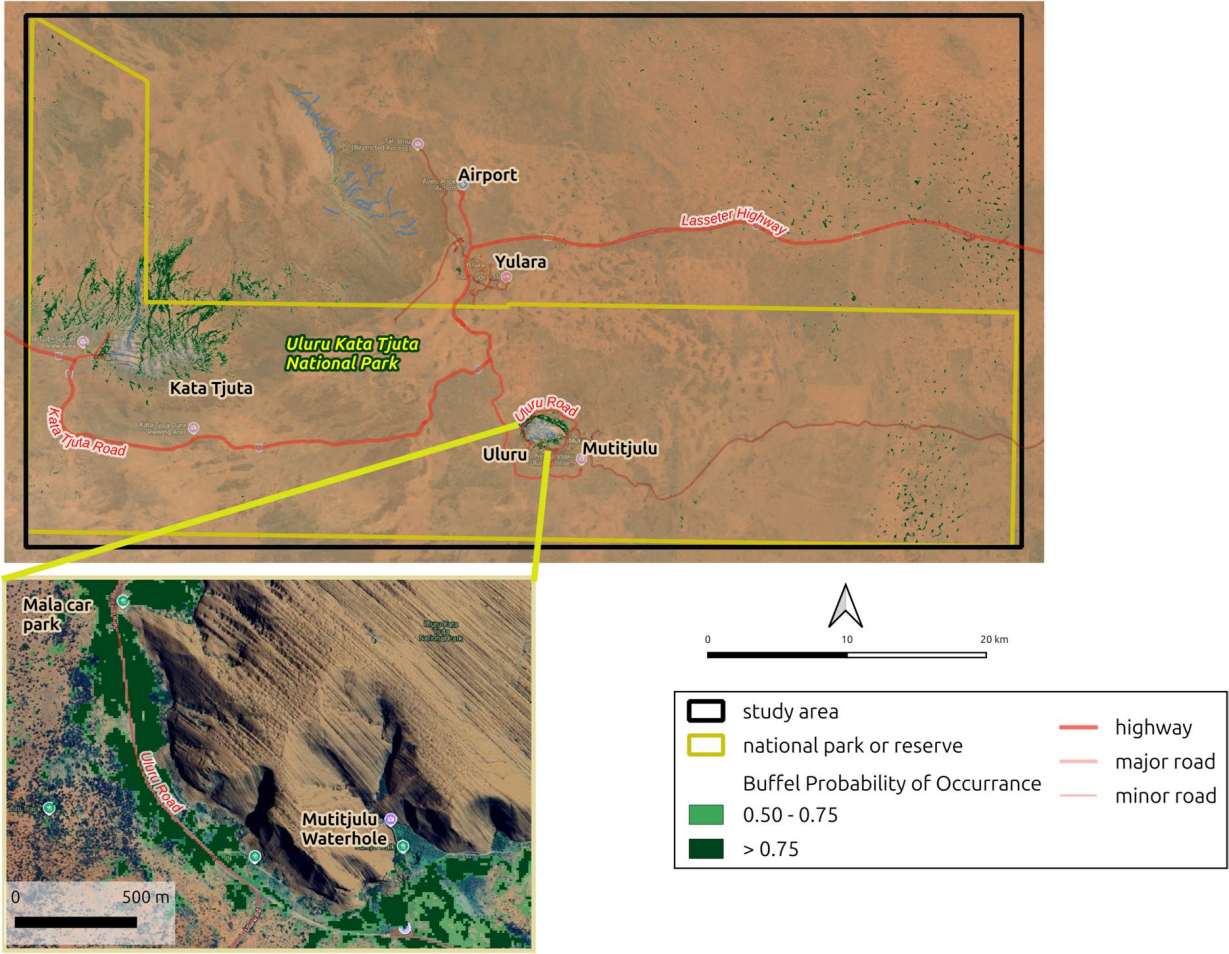
Buffel grass probability map for Uluru-Kata Tjuta National Park (UKTNP). Imagery © 2026 Google Earth

Logistic regression models are most useful when they minimise the number of false positives (classifying a buffel grass-free waypoint as a buffel grass waypoint) and false negatives (buffel grass waypoints called buffel grass-free waypoints). In our study, a probability threshold of 0.50 for UKTNP and 0.52 for TWMNP provided the optimal trade-off for each study area, respectively. Therefore, a probability threshold of 0.5 was used to create a thematic map of buffel occurrence in both TWMNP and UKTNP (Figs. 3 and 4).

### Buffel grass probability estimates within landscape classes

We collected waypoints from five of the eight landscape classes in TWMNP, and two of the three landscape classes in UKTNP. Similar to the overall models for both study areas, the average model accuracy across landscape classes was 84% for TWMNP and 87% for UKTNP (Table 4), suggesting that the models were able to accurately identify buffel grass from buffel grass-free areas within and across landscape types.

**Table 4:** Model specificity, sensitivity and accuracy within landscape classes for Tjoritja/West MacDonnell National Park (TWMNP) and Uluru-Kata Tjuta National Park (UKTNP). The average model accuracy was 84% for TWMNP and 87% for UKTNP, similar to the overall modelling results for both study areas

Study area	Waypoints (#)	Area (km^2^)	Land system	Class	Specificity (%)	Sensitivity (%)	Accuracy (%)
TWMNP	138	261	Bond springs	Granite hills	91.89	82.81	0.877
	216	493	Gillen	Sandstone ranges	83.84	85.47	0.847
	85	1505	Harts	Granite ranges	68.97	83.93	0.788
	9	33	Pertnjara	Limestone hills	66.67	100	0.778
	32	94	Todd	Alluvial floodplains	88.89	92.86	0.906
UKTNP	110	2523	Simpson	Desert dunefields	94.51	78.95	0.918
	74	202	Gillen	Sandstone ranges	77.27	84.62	0.824

The modelled probability of buffel grass occurrence (i.e. probability ≥ 50%) varied among landscape classes (Table 5). In the TWMNP study area, buffel grass was predicted to occur in over 87% of alluvial floodplains landscape classes, but in only 22% of limestone hills, with all other landscape classes having intermediate values. In the UKTNP study area, the probability of buffel grass occurrence was < 20% in all three landscape classes. Although desert dunefields constitute about 93% of the study area, the probability of buffel grass occurrence was only 1%.

**Table 5:** Probability of buffel grass occurrence using the 50% (p50) and 75% (p75) thresholds for Tjoritja/West MacDonnell National Park (TWMNP) and Uluru-Kata Tjuta National Park (UKTNP). Area refers to the total size (and percentage) of the landscape class within the study area

Study area	Class	Area (km^2^)	Area (%)	p50	p75	Land systems
TWMNP	Alluvial floodplains	209	8	0.87	0.70	Bushy Park, Hamilton, Todd
TWMNP	Desert sandplains	42	2	0.68	0.49	Ewaninga
TWMNP	Granite hills	267	10	0.66	0.46	Bond Springs
TWMNP	Granite ranges	1541	55	0.65	0.43	Harts
TWMNP	Limestone plains and rises	35	1	0.50	0.31	Muller
TWMNP	Granite plains and rises	25	1	0.41	0.24	Stokes
TWMNP	Sandstone ranges	674	24	0.41	0.27	Gillen, Sonder
TWMNP	Limestone hills	66	2	0.22	0.12	Huckitta, Pertnjara
UKTNP	Sandstone ranges	175	6	0.18	0.11	Gillen
UKTNP	Desert dunefields	2544	93	0.01	0.00	Simpson
UKTNP	Sandstone hills	24	1	0.00	0.00	Kernot

## Discussion

### Overall model performance

In both study areas, the models were able to accurately distinguish buffel grass from buffel grass-free areas (i.e. 85% for TWMNP and 94% for UKTNP). Specific to mapping buffel grass using satellite imagery, our model accuracies are higher than previous attempts to map buffel grass in the arid zone of Australia (e.g. Marshall et al., 2012) and in North America using MODIS and Landsat image data (e.g. Wallace et al., 2016). Our model accuracies are comparable to those reported in North America by Elkind et al. (2019) using Worldview-2 satellite data. The comparable accuracies for this study are notable because the Sentinel-2 image data used in our study are routinely captured, freely available and have 10-m spatial resolution. The WorldView-2 image data feature a higher spatial resolution (2 m) but have a limited footprint and tasking is required, which results in a limited image archive.

Central Australia is characterised by variable (both temporally and spatially) rainfall, long-lasting fire scars, a highly variable occurrence of bare ground, and highly variable ground and overstorey coverage (more so in TWMNP than UKTNP)—all which contribute to the spectral reflectance of a pixel. The advantages of using temporal information captured within satellite archives for mapping and monitoring ecosystems have been widely reported (e.g. Pasquarella et al., 2016). The unique phenology of buffel grass makes it possible to distinguish it from other vegetation types using satellite imagery, especially when its behaviour can be tracked over both wet and dry periods. Thus, the use of a multi-year time series to isolate the phenological signal of buffel grass, compared to native vegetation, likely contributed to our high model accuracies. Badreldin et al. (2021) reported similar accuracies for mapping three grassland classes (native, exotic and mixed) in a semiarid area of Saskatchewan, Canada, utilising a large multispectral and multitemporal dataset and data dimensionality reduction techniques. More recently, Tiwari et al. (2024) proposed that using a time series of vegetation indices could serve as an early indicator of weed infestation in standing crops. Our results indicate that a multi-year time series of Sentinel-2 data effectively captured the distinct phenology and signature of buffel grass despite its occurrence within a highly variable (both temporally and spatially) landscape.

The buffel grass maps of our two study areas encompass approximately 5595 km^2^ and utilised over sixteen billion data points (number of pixels *x* number of satellite images) for each vegetation index (over 64 billion points in total). This study demonstrates that recent advances in the computation power of widely available ‘gaming’ computers, as used in this study, and time series of satellite imagery can be combined to produce accurate maps of buffel grass occurrence in central Australia. Computing eigenvector values via the SVDs was the most computationally intensive part of the process, a process that was entirely tractable with a desktop PC for the given area. Our study method used widely available open-source data and algorithms and demonstrates the potential to map buffel grass occurrence across large areas rapidly and at minimal cost compared to methods using aerial surveys and/or drones.

### Spatial occurrence of buffel grass

The spatial occurrence of buffel grass differed between the two study areas, which is not surprising as TWMNP is a mosaic of varying landscape classes, while extensive dunefields and irregular sand dunes dominate the UKTNP landscape. In the TWMNP study area, model results were closely aligned with our current understanding of the environmental preferences of buffel grass. For example, within the TWMNP five of the eight landscape classes had modelled buffel grass infestation of 50% or more, and these land types have relatively high nutrient and medium textured soils which are favourable to buffel grass establishment. Our model results suggest the more productive land types with deep alluvial soils and periodically flooded drainage lines and/or plains were likely to contain buffel grass. Conversely, model results suggest that buffel grass occurrence in TWMNP was low in the less productive land types characterised by sandstone/quartzite ranges, limestone hills, shallow rocky soils and/or extensive bare rock. Not surprisingly, buffel grass infestation was also highest in vegetation types that are associated with higher resource alluvial soils, such as *Acacia* shrublands and woodlands with tussock grass understories. Conversely, vegetation types with open canopies and a hummock grassland ground layer had relatively low modelled buffel grass infestation and were correlated with the less productive land types described above.

Within the UKTNP study area, the modelled buffel grass infestation was relatively straightforward and contrasted with the spatial pattern of buffel infestation in TWMNP. Specifically, 93% of the UKTNP study area is dominated by a sand plain/dunefield land type, yet only 1% of the study area was predicted to contain buffel grass. In contrast, sandstone ranges cover about 6% of the study area but our probability map predicted buffel grass occurs across nearly 20% of this land type. These spatial patterns align closely with the known distribution of buffel grass in UKTNP, with the highest concentrations of buffel grass occurring in the run-on sites around the base of Uluru and Kata Tjuta. Although vegetation mapping for the UKTNP study area is too coarse for a detailed pattern analysis, the modelled buffel grass concentrations confirm the known close association of buffel grass with *Corymbia* and *Eucalyptus* woodland habitats that occur near the bases of Uluru and Kata Tjuta.

Across both study areas, the modelled spatial occurrence of buffel grass closely aligned with existing knowledge of its habitat preferences (Lawson et al., 2004) and historical establishment across the region (Puckey & Albrecht, 2004). This suggests that using local knowledge of landforms and vegetation types could increase the efficiency of management and control actions by focusing on areas where buffel grass is most likely to be present, even if those areas have yet to be mapped. Alternatively, management may instead focus on maintaining low buffel grass cover in habitats that have so far withstood incursion but are now under increasing invasion pressure.

### Applicability to other regions worldwide

This study was conducted in central Australia and in consideration of the irregular spatial and temporal rainfall patterns characteristic of the region. However, there is nothing in the conceptualisation or implementation of our technique that would limit its application to other regions where buffel incursions occur, both inside and outside of Australia, including areas with predictable seasonal rainfall. For example, our technique was used to successfully map both GDEs and the highly invasive weed, gamba grass (*Andropogon gayanus*), in the Australian tropical savanna biome (Box and Brim Box 2025), an area with predictable wet and dry seasons. Buffel grass was successfully mapped in small areas in Australia (Marshall et al., 2012) and North America (Wallace et al., 2016) based on its response to rainfall (timing and greenness), and we expect these same areas could be mapped scaling up to a much larger area using our technique. In addition, our technique should also be applicable to any species that has a detectable and unique phenological signature, as buffel grass has in our example.

### Limitations and future research

The limitations to effectively map buffel grass in central Australia are minimal but do occur. If the overstorey coverage is dense, the satellite will most likely detect the spectral signal of the canopy and not the ground cover. However, in central Australia, overstorey coverage is spatially limited, so if buffel is present in the area surrounding trees, it will be captured in those pixels, and an inference could be made that buffel may be present under canopy trees.

While the imagery can be collated and processed in a relatively short amount of time, the results cannot be reliably interpreted or quantified without field verification and calibration. We have made no attempt to recommend minimal numbers of waypoints required, but if too few waypoints are collected, buffel grass can be confused with native grasses. For example, in the UKTNP study area, 40% of buffel grass-free waypoints collected from resource-rich swales on the eastern side of the park were misclassified as containing buffel grass. These sites consisted mainly of native grasses. Given only 10 waypoints were collected in this unique land type, collecting additional waypoints to ‘train’ the model may eliminate these types of misclassifications. Future research could better ascertain the minimal number of waypoints needed to retain model accuracy while minimising the amount of ground-truthing needed. Interpretations with no field verification should not be considered reliable.

A potential avenue for future research is to link the temporal information from the training and validation data (waypoints) to the image data time series. If a waypoint is surveyed on multiple occasions, knowledge of buffel grass presence and ground cover may enable fine-tuning of the model. A waypoint may at one point in time be buffel grass-free, then be the location of a new invasion front, before again being buffel grass-free due to direct management actions. Incorporating such information into the methodology may tease out otherwise cryptic signals in the time series data.

Given the success of our modelling efforts here, it is reasonable to use these results to investigate future steps to manage current buffel incursions and to simulate future incursions. Weed spread, as well as costs around weed control, have been successfully modelled in other areas (e.g. Aurambout & Endress, 2018). The computing power and recent availability of multi-agent programmable modelling environments have lessened the problems associated with complex modelling using, for example, cellular automaton (McCoy et al., 2003). Going forward, much can be done to further our understanding of the rates at which buffel spreads, where it is most likely to spread and how best to use limited resources to mitigate the negative consequences of buffel incursions.

## Conclusions

The application of a SVD to a time series of vegetation indices allowed us to map buffel grass occurrence across a large area of central Australia (over 5600 km^2^) with high accuracy (≥ 85%). Because our method uses free, open-source data sets and software, it is more cost-effective than other methods commonly used to map buffel grass, including drones and/or aerial surveys. We caution, however, that our methodology has not been widely tested in other regions, both within and outside of Australia.

## Data Availability

Data generated in this project are located within the Department of Lands, Planning and Environment’s spatial library and are available on reasonable request.
